# Numerical Characterization of DNA Sequence Based on Dinucleotides

**DOI:** 10.1100/2012/104269

**Published:** 2012-04-24

**Authors:** Xingqin Qi, Edgar Fuller, Qin Wu, Cun-Quan Zhang

**Affiliations:** ^1^School of Mathematics and Statistics, Shandong University at Weihai, Weihai 264209, China; ^2^Department of Mathematics, West Virginia University, Morgantown, WV 26506, USA; ^3^School of IOT Engineering, Jiangnan University, Wuxi 214122, China

## Abstract

Sequence comparison is a primary technique for the analysis of DNA sequences. In order to make quantitative comparisons, one devises mathematical descriptors that capture the essence of the base composition and distribution of the sequence. Alignment methods and graphical techniques (where each sequence is represented by a curve in high-dimension Euclidean space) have been used popularly for a long time. In this contribution we will introduce a new nongraphical and nonalignment approach based on the frequencies of the dinucleotide *XY* in DNA sequences. The most important feature of this method is that it not only identifies adjacent *XY* pairs but also nonadjacent *XY* ones where *X* and *Y* are separated by some number of nucleotides. This methodology preserves information in DNA sequence that is ignored by other methods. We test our method on the coding regions of exon-1 of *β*–globin for 11 species, and the utility of this new method is demonstrated.

## 1. Introduction

 The number of identifiable DNA sequences responsible for various physiological structures is rapidly increasing as more and more collected DNA sequences are added to scientific databases. It is, however, difficult to obtain information directly from sequences since the sheer volume of data is computational demanding. It is one of the challenges for biologists to analyze mathematically the large volume of genomic DNA sequence data. Many schemes have been proposed to numerically characterize DNA sequences.

Sequence alignment has been used as a very powerful tool for comparison of two closely related genomes at the base-by-base nucleotide sequence level. This method relies heavily on the orderings of nucleotides appearing in the sequence. With the divergence of species over time, though, genomic rearrangements and in particular genetic shuffling make sequence alignment unreliable or impossible.

Graphical techniques are another powerful tool for the analysis and visualization of DNA sequences. Using graphical approaches can provide intuitive pictures or useful insights that assist the analysis of complicated relations between DNA sequences. This methodology starts with a graphical representation of DNA sequence which could be based on 2D, 3D, 4D, 5D, and 6D spaces and represents DNA as matrices by associating with the selected geometrical objects, then vectors composed of the invariants of matrices will be used to compare DNA sequences, see [1–10]. Such schemes have an advantage in that they offer an instant, though, visual and qualitative summary of the lengthy DNA sequences. This approach also involves many unresolved questions. For example, how does one obtain suitable matrices to characterize DNA sequences and how are invariants selected suitable for sequence comparisons? In many cases, the calculation of the matrices or the invariants will become more and more difficult with the length of the sequence. There are also approaches which could arrive a mathematical representation of DNA sequences by nongraphical ways, see [11–13]. And more recently, a new representation based on symbolic dynamics [14] and a new representation based on digital signal method [15] are also illustrated.

In this contribution, we introduce a novel nongraphical and nonalignment approach for DNA sequence comparison. We use DNA sequence directly by considering the frequencies of dinucleotide. We represent each DNA sequence by a dinucleotide frequency matrix or by a dinucleotide frequency vector, based on which two distance measurements are defined, respectively. Then comparisons between DNA sequences could be carried out by calculating the distances between these mathematical descriptors. The most important feature of this method is that the mathematical descriptors not only take into consideration the frequencies of adjacent *XY* pairs but also of nonadjacent *XY* pairs. In this way, information contained in the relative spacing of nucleotides is preserved. The method is very simple and fast, and does not require sequence alignment or sequence graphical representation which would cause complex calculations. It can be used to analyze both short and long DNA sequences. As an application, this method is tested on the exon-1 coding sequences of *β*-globin for 11 species and the results are consistent with what have been reported previously [5, 9, 12, 14, 15], which prove the utility of this new method.

## 2. Dinucleotide Frequency Matrix and Dinucleotide Frequency Vector

Typically, DNA sequence data is represented as a string of letters A, C, G, and T, which signify the four nucleotides: adenine, cytosine, guanine, and thymine, respectively. There are 16 possible dinucleotides, that is, Ω = { AT, AA, AC, AG, TT, TA, TC, TG, GT, GA, GC, GG, CT, CA, CC, CG}. In the following, we always use *XY* to represent dinucleotides, and note that dinucleotide *XY* is distinguished from.

Let *s* be a sequence of length *n* and denote the number of occurrences of adjacent *XY* in *s* by *Y* ^(1)^. Clearly, if *s* is a sequence of length, then ∑_*XY*∈*Ω*_ *XY* ^(1)^ = *n* − 1. The occurrence frequency for *XY* is defined as

(1) $$f_{XY}^{\left(1\right)}=\frac{XY^{\left(1\right)}}{\left(n-1\right)}.$$

We get one 16-dimensional vector ${\hat{f}}^{\left(1\right)}$ associated with sequence *s* based on adjacent dinucleotides:

(2) $${\hat{f}}^{\left(1\right)}=\left(f_{\text{AT}}^{\left(1\right)},f_{\text{AA}}^{\left(1\right)},f_{\mathrm{AC}}^{\left(1\right)},\ldots ,f_{\text{CT}}^{\left(1\right)},f_{\text{CA}}^{\left(1\right)},f_{\text{CC}}^{\left(1\right)},f_{\text{CG}}^{\left(1\right)}\right).$$

Notice that there would be a loss of information when one condenses sequence *s* to a single 16-dimensional vector. A way to recover some of the lost information associated with a sequence *s* to a single 16-vector is to introduce additional 16 vectors to store the frequency information of pairs *XY* when *X* and *Y* are not adjacent but are separated at various distance. For example, if *s* = ATCGATC, the *adjacent* dinucleotides are AT, TC, CG, GA with occurrence frequency 2/6, 2/6, 1/6, and 1/6, respectively. The dinucleotides *at distance *2 (i.e., separated by one nucleotide) in *s* are AC, TG, CA, GT, AC with occurrence frequency 2/5, 1/5, 1/5, and 1/5, respectively. These two 16-dimensional vectors will contain additional information beyond that found in the initial dinucleotide vector.

Generally, let *s* be a sequence of length. Denote *XY* ^(*d*)^ as the number of occurrence of *XY* in *s* when *X* and *Y* are separated by *d* − 1 nucleotides. Clearly, ∑_*XY*∈*Ω*_ *XY* ^(*d*)^ = *n* − *d*. Define

(3) $$f_{XY}^{\left(d\right)}=\frac{XY^{\left(d\right)}}{\left(n-d\right)},$$

as the occurrence frequency. For each given integer, we could get one 16-dimensional vector ${\hat{f}}^{\left(d\right)}$ associated with sequence *s*:

(4) $${\hat{f}}^{\left(d\right)}=\left(f_{\text{AT}}^{\left(d\right)},f_{\text{AA}}^{\left(d\right)},f_{\mathrm{AC}}^{\left(d\right)},\ldots ,f_{\text{CT}}^{\left(d\right)},f_{\text{CA}}^{\left(d\right)},f_{\text{CC}}^{\left(d\right)},f_{\text{CG}}^{\left(d\right)}\right).$$

The distance *d* between *X* and *Y* could be 1, 2 or even larger integers. When we scan sequence *s* to count the occurrence of dinucleotides *XY* at distance, the nucleotides of *s* from position 1 to (*n* − *d*) are counted as “*X*”, while the nucleotides of *s* from position (*d* + 1) to *n* are counted as “*Y*”. When *d* ≤ ⌊(*n* − 1)/2⌋, there is an overlapping interval [*d* + 1, *n* − *d*] between the two intervals [1, *n* − *d*] and [*d* + 1, *n*], which means the nucleotides in the overlapping interval will counted as both *X* and *Y*; but if *d* > ⌈(*n* − 1)/2⌉, the two intervals [1, *n* − *d*] and [*d* + 1, *n*] will disjoint, and the information of these nucleotides in the interval [*n* − *d* + 1, *d*] will be lost. So in the following, to avoid loss of information, *d* must not be larger than ⌈(*n* − 1)/2⌉, that is, *d* ≤ ⌈(*n* − 1)/2⌉. Furthermore, to make the information in ${\hat{f}}^{\left(d\right)}$ more accurate, we hope that the overlapping interval [*d* + 1, *n* − *d*] will be large enough. Based on this intuition, we would prefer to these *d* such that (*n* − 2*d*)/*n* ≥ 50%, which guarantees that more than half of the nucleotides in sequence *s* will be counted as both *X* and *Y*. So *d* is restricted to *d* ≤ ⌊*n*/4⌋ for each DNA sequence *s* with length.

Let *s* be a DNA sequence of length, for a given *d* ≤ ⌊*n*/4⌋, the *dinucleotide frequency matrix* associated with *s* is defined as

(5) F(s)=(f^(1)f^(2)f^(3)⋮f^(d)),

where ${\hat{f}}^{\left(i\right)}$ is the 16-dimensional occurrence frequency vector when *X* and *Y* are separated by (*i* − 1) nucleotides. The size of matrix *F*(*s*) is *d* × 16.

We also present another mathematical descriptor associated with *s* named *dinucleotide frequency vector* which is defined as

(6) $$\hat{F}\left(s\right)=\left({\hat{f}}^{\left(1\right)},{\hat{f}}^{\left(2\right)},{\hat{f}}^{\left(3\right)},\ldots ,{\hat{f}}^{\left(d\right)}\right),$$

then $\hat{F}(s)$ is a 1 × 16*d* row vector.

## 3. Two Distance Measurements Based on Dinucleotide Frequency

 From Section 2, we get correspondences between one DNA sequence *s* and the dinucleotide frequency matrix *F*(*s*) and the dinucleotide frequency vector $\hat{F}\left(s\right)$. Note that the sizes of *F*(*s*) and $\hat{F}\left(s\right)$ all depend on. To make the comparisons for a set of DNA sequences meaningful, we should use an identical *d* for all these DNA sequences. Denote the set of DNA sequences by, by the discussion in Section 2, we define the identical *d*_0_ as

(7) $$d_{0}=\underset{s\in S}{\min}\left\lfloor \frac{\left(\left|s\right|\right)}{4}\right\rfloor ,$$

where |*s*| is the length of *s*. The choice of *d*_0_ will guarantee that either the frequency matrix or the frequency vector will involve enough accurate information, and the dinucleotide frequency matrices and dinucleotide frequency vectors associated with sequences in *S* all have the same size. DNA sequences comparisons could be completed by studying their corresponding matrices and vectors. In the following we will introduce two different distance measurements based on dinucleotide frequencies matrix and dinucleotide frequency vector, respectively.

### 3.1. City Block Distance for Dinucleotide Frequency Matrix

 Given two DNA sequences *s* and *h*, then we get the dinucleotide frequency matrix *F*(*s*) and *F*(*h*) as in Section 2, comparison between *s* and *h* becomes comparison between *F*(*s*) and *F*(*h*). Using this, we define the city block distance *d*_1_(*s*, *h*) between *s* and *h* as

(8) $$d_{1}\left(s,h\right)=\underset{1\leq i\leq d_{0},\;\;1\leq j\leq 16}{\sum }\left|F_{ij}\left(s\right)-F_{ij}\left(h\right)\right|.$$

### 3.2. Cosine Distance for Dinucleotide Frequency Vector

We also obtain a mapping from a DNA sequence *s* to a vector $\hat{F}\left(s\right)$ in the 16*d*_0_-dimensional linear space. Comparison between DNA sequences also could become comparison between these 16*d*_0_-dimensional vectors. This is based on the assumption that two DNA sequences are similar if the corresponding 16*d*_0_-dimensional vectors in the 16*d*_0_-dimensional space have similar directions. Given two DNA sequences *s* and *h*, the dinucleotide frequency vectors are $\hat{F}\left(s\right)$ and $\hat{F}\left(h\right)$, we define the cosine distance *d*_2_(*s*, *h*) between *s* and *h* as

(9) $$d_{2}\left(s,h\right)=1-\cos\left(\hat{F}\left(s\right),\hat{F}\left(h\right)\right),$$

where $\cos\left(\hat{F}\left(s\right),\hat{F}\left(h\right)\right)$ is the cosine value of the included angle between vectors $\hat{F}\left(s\right)$ and $\hat{F}\left(h\right)$.

## 4. Applications and Experimental Results

### 4.1. Experimental Results

 A comparison between a pair of DNA sequences to judge their similarity or dissimilarity could be carried out by calculating the distance *d*_1_(*s*, *h*) or *d*_2_(*s*, *h*). The smaller is the distance, the much similar are the two DNA sequences (The code is available on request).

To test the utility of above method, we make a comparison for the coding regions of exon-1 of *β*-globin gene for 11 different species, which were also studied by Randić et al. in [12].  Table 1 presents their accession numbers in NCBI database, while Table 2 lists these 11 coding sequences concretely.

At first, we present the similarity/dissimilarity matrix based on distance measurement *d*_1_, see Table 3. When we examine this table, we notice that smallest entries are always associated with the pairs (human, chimpanzee) with *d*_1_ = 2.5567, (human, gorilla) with *d*_1_ = 2.4026, and (gorilla, chimpanzee) with *d*_1_ = 2.7338. That means the more similar species pairs are human-gorilla, human-chimpanzee, and gorilla-chimpanzee. We also observe that the largest entry *d*_1_ = 9.0347 is associated with gallus and lemur and the larger entries appear in the rows belonging to gallus and opossum, which is consistent with the facts that gallus is the only nonmammalian species among these 11 species and opossum is the most remote species from the remaining mammals. These observed facts are consistent with the results reported in previous studies [5, 9, 12] determined by matrix invariants techniques, and also consistent with the reported results from nongraphical means [14, 15]. More interesting, in Table 3, the distance between goat and bovine is *d*_1_ = 2.3438, which is actually the smallest entry in Table 3. That implies goat and bovine are regarded to be much similar to each other by our method, which is consistent with their biology taxonomy that bovine and goat are both even-toed ungulates and belong to the family of “Bovidae”.

Table 4 presents the similarity/dissimilarity matrix based on the distance measurement *d*_2_. The smallest entries are also associated with the pairs (human, chimpanzee) with *d*_2_ = 0.0087, (human, gorilla), with *d*_2_ = 0.0074, and (gorilla, chimpanzee), and with *d*_2_ = 0.0112. We find that the largest entry (*d*_2_ = 0.1139 ) is associated with (gallus, lemur), and the rows corresponding to gallus and opossum have larger entries, which is also consistent with the facts that gallus is the only nonmammalian species among these 11 species and opossum is the most remote species from the remaining mammals. The observed facts in Table 4 are consistent with the previously reported results in [5, 9, 12, 14, 15] as well. And the distance between goat and bovine (*d*_2_ = 0.0109 ) is also much smaller as we expect.

**Figure 1:**
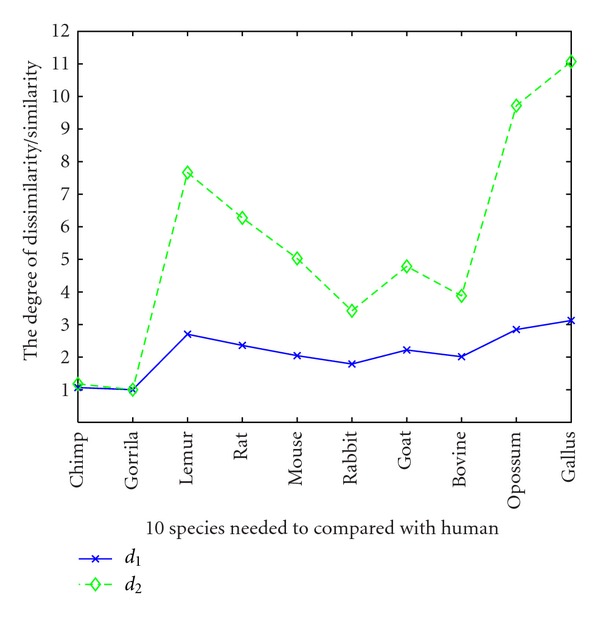
The degree of dissimilarity/similarity of the other 10 species with human, where the degree of dissimilarity/similarity of the pair human-gorilla is defined relatively as 1.

We can see that there is an overall qualitative agreement between Tables 3 and 4. To see it visually, we denote the degree of dissimilarity/similarity of the pair human-gorilla as 1 in each table, then the results of the examination of the degree of dissimilarity/similarity between human and other several species under the two distance measurements are shown in Figure 1. We can see that the curvilinear trend of these two curves are almost the same, which demonstrates the overall agreement among dissimilarity/similarities obtained by these two distance methods.

### 4.2. Discussion

**Figure 2:**
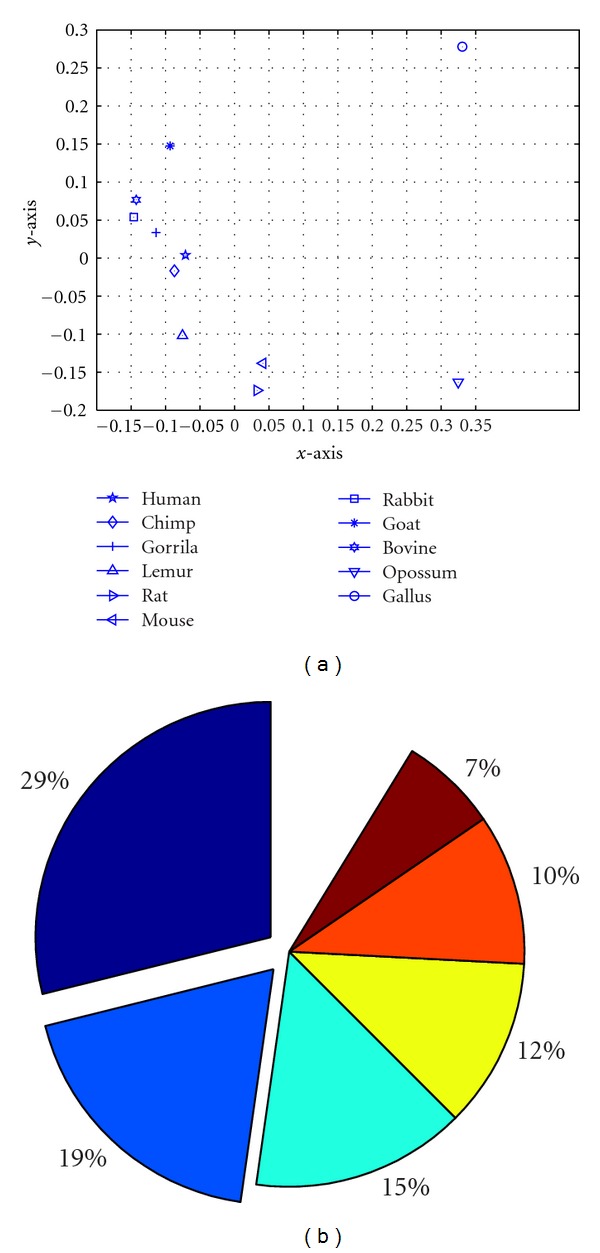
(a) The projection of the 336-dimensional vectors of 11 species on a 2D space composed of the top two principal components; (b) The contributions of the first 6 principal components.

For the above exon-1 coding data of 11 species, *d*_0_ is chosen to be 21 followed by (7). A 336-dimensional vector is used to characterize each DNA sequence under the second distance measure. To confirm the efficacy of the vectors constructed in this high-dimensional data representation, we perform principal component analysis (PCA) on these 336 parameters.  Figure 2(a) shows the projection of the 11 vectors on a 2D property space composed of the top two principal components PC1, PC2. We can see that in the 2D space, gallus (labeled by “⨀”) and opossum (labeled by “∇”) are furthest from the other 9 species, and human, chimpanzee, and gorilla are very close to each other. These result are consistent with what we have got from Table 4. Note that these top two principal components contribute 48% (see Figure 2(b)) to the total information. Some information is lost when we do the projection, for example, bovine seems much closer to rabbit than goat in the 2D projection, but we know this is not true in Table 4 when all 336 parameters are considered. However, this rough approximation confirms that our mathematical descriptor characterizes DNA sequence structure effectively.

## 5. Conclusion

 In this paper, we have presented a new method based on dinucleotide frequencies for DNA sequence comparison. The dinucleotide frequency matrix and dinucleotide frequency vector are used to mathematically characterize a DNA sequence. The most important feature of this method is that the mathematical descriptors not only involve the frequencies of adjacent *XY* pairs but also nonadjacent *XY* pairs (i.e., when *X* and *Y* are separated by various number of nucleotides), such that a lot of important information is avoided to lose. This new method does not require sequence alignment or sequence graphical representation, which avoids the complex calculation found in either sequence alignment or sequence graphical representation. The method is very simple and fast, and it can be used to analyze both short and long DNA sequences with high efficiencies.

## Figures and Tables

**Table 1:** ID Information for Exon-1 of *β* -globin gene of 11 species.

Species	ID/Accession	Database	length
Human	U01317	NCBI	92
Chimpanzee	X02345	NCBI	105
Gorilla	X61109	NCBI	93
Lemur	M15734	NCBI	92
Rat	X06701	NCBI	92
Mouse	V00722	NCBI	93
Rabbit	V00882	NCBI	92
Goat	M15387	NCBI	86
Bovine	X00376	NCBI	86
Opossum	J03643	NCBI	92
Gallus	V00409	NCBI	92

**Table 2:** The coding sequence of exon-1 of *β* -globin gene for 11 species.

Species	DNA sequence
Human	ATGGTGCACCTGACTCCTGAGGAGAAGTCTGCCGTTACTGCCCTGTGGGGCAAGGTGAACGTGGATGAAGTTGGTGGTGAGGCCCTGGGCAG
Chimpanzee	ATGGTGCACCTGACTCCTGAGGAGAAGTCTGCCGTTACTGCCCTGTGGGGCAAGGTGAACGTGGATGAAGTTGGTGGTGAGGCCCTGGGCAGGTTGGTATCAAGG
Gorilla	ATGGTGCACCTGACTCCTGAGGAGAAGTCTGCCGTTACTGCCCTGTGGGGCAAGGTGAACGTGGATGAAGTTGGTGGTGAGGCCCTGGGCAGG
Lemur	ATGACTTTGCTGAGTGCTGAGGAGAATGCTCATGTCACCTCTCTGTGGGGCAAGGTGGATGTAGAGAAAGTTGGTGGCGAGGCCTTGGGCAG
Rat	ATGGTGCACCTAACTGATGCTGAGAAGGCTACTGTTAGTGGCCTGTGGGGAAAGGTGAACCCTGATAATGTTGGCGCTGAGGCCCTGGGCAG
Mouse	ATGGTTGCACCTGACTGATGCTGAGAAGTCTGCTGTCTCTTGCCTGTGGGCAAAGGTGAACCCCGATGAAGTTGGTGGTGAGGCCCTGGGCAGG
Rabbit	ATGGTGCATCTGTCCAGTGAGGAGAAGTCTGCGGTCACTGCCCTGTGGGGCAAGGTGAATGTGGAAGAAGTTGGTGGTGAGGCCCTGGGCAG
Goat	ATGCTGACTGCTGAGGAGAAGGCTGCCGTCACCGGCTTCTGGGGCAAGGTGAAAGTGGATGAAGTTGGTGCTGAGGCCCTGGGCAG
Bovine	ATGCTGACTGCTGAGGAGAAGGCTGCCGTCACCGCCTTTTGGGGCAAGGTGAAAGTGGATGAAGTTGGTGGTGAGGCCCTGGGCAG
Opossum	ATGGTGCACTTGACTTCTGAGGAGAAGAACTGCATCACTACCATCTGGTCTAAGGTGCAGGTTGACCAGACTGGTGGTGAGGCCCTTGGCAG
Gallus	ATGGTGCACTGGACTGCTGAGGAGAAGCAGCTCATCACCGGCCTCTGGGGCAAGGTCAATGTGGCCGAATGTGGGGCCGAAGCCCTGGCCAG

**Table 3:** The upper triangular part of the dissimilarity/similarity matrix based on *d*_1_.

Species	Human	Chimpanzee	Gorilla	Lemur	Rat	Mouse	Rabbit	Goat	Bovine	Opossum	Gallus
Human	0	2.5567	2.4026	6.4922	5.6622	4.9144	4.2904	5.3220	4.8306	6.8358	7.4959
Chimpanzee		0	2.7338	6.5340	5.9455	5.1613	4.9587	5.6525	4.9670	7.4568	7.9791
Gorilla			0	7.0466	6.2344	5.2819	5.0310	5.3353	4.9340	7.8956	8.0582
Lemur				0	6.9735	6.8419	5.6647	6.9332	6.0195	8.2293	9.0347
Rat					0	5.2540	6.8004	6.5847	6.2545	7.5359	8.2347
Mouse						0	6.5730	6.7863	6.4133	7.2900	7.8317
Rabbit							0	5.9265	5.2974	8.0743	8.3210
Goat								0	2.3438	8.0158	7.7129
Bovine									0	7.9847	8.2938
Opossum										0	8.0268
Gallus											0

**Table 4:** The upper triangular part of the dissimilarity/similarity matrix based on *d*_2_.

Species	Human	Chimpanzee	Gorilla	Lemur	Rat	Mouse	Rabbit	Goat	Bovine	Opossum	Gallus
Human	0	0.0087	0.0074	0.0567	0.0464	0.0372	0.0253	0.0354	0.0287	0.0719	0.0819
Chimpanzee		0	0.0112	0.0564	0.0487	0.0383	0.0303	0.0403	0.0320	0.0793	0.0899
Gorilla			0	0.0619	0.0538	0.0398	0.0312	0.0357	0.0302	0.0887	0.0877
Lemur				0	0.0691	0.0635	0.0454	0.0616	0.0463	0.0939	0.1139
Rat					0	0.0417	0.0631	0.0592	0.0552	0.0832	0.1048
Mouse						0	0.0588	0.0573	0.0528	0.0765	0.0932
Rabbit							0	0.0444	0.0349	0.0998	0.0933
Goat								0	0.0109	0.0948	0.0792
Bovine									0	0.0923	0.0937
Opossum										0	0.0897
Gallus											0
